# Alkaloids: Therapeutic Potential against Human Coronaviruses

**DOI:** 10.3390/molecules25235496

**Published:** 2020-11-24

**Authors:** Burtram C. Fielding, Carlos da Silva Maia Bezerra Filho, Nasser S. M. Ismail, Damião Pergentino de Sousa

**Affiliations:** 1 Molecular Biology and Virology Research Laboratory, Department of Medical Biosciences, University of the Western Cape, Bellville 7535, South Africa; bfielding@uwc.ac.za; 2 Department of Pharmaceutical Sciences, Federal University of Paraíba, Paraíba 58051-900, Brazil; carlosmaia1996@gmail.com; 3 Pharmaceutical Chemistry Department, Faculty of Pharmaceutical Sciences and Pharmaceutical Industries, Future University in Egypt, Cairo 12311, Egypt; saadnasser2003@yahoo.com

**Keywords:** COVID-19, natural products, antiviral drug, SARS-CoV, MERS-CoV, coronaviruses, virus, SARS-CoV-2

## Abstract

Alkaloids are a class of natural products known to have wide pharmacological activity and have great potential for the development of new drugs to treat a wide array of pathologies. Some alkaloids have antiviral activity and/or have been used as prototypes in the development of synthetic antiviral drugs. In this study, eleven anti-coronavirus alkaloids were identified from the scientific literature and their potential therapeutic value against severe acute respiratory syndrome coronavirus-2 (SARS-CoV-2) is discussed. In this study, in silico studies showed an affinity of the alkaloids for binding to the receptor-binding domain of the SARS-CoV-2 spike protein, putatively preventing it from binding to the host cell. Lastly, several mechanisms for the known anti-coronavirus activity of alkaloids were discussed, showing that the alkaloids are interesting compounds with potential use as bioactive agents against SARS-CoV-2.

## 1. Introduction

Current drug recommendations for the treatment of coronavirus disease-2019 (COVID-19) are based on historical reports from various severe acute respiratory syndrome coronavirus (SARS-CoV) and Middle Eastern respiratory syndrome coronavirus (MERS-CoV) studies [1]. Some evidence from these studies suggests that the use of an integrative approach, such as the use of western medicine with herbal medicines and/or medicinal plant-based natural compounds, is more effective in controlling the coronavirus infection and in reducing the number of deaths [2]. Also, the use of effective combinational therapy could reduce the effective concentration of compounds below the therapeutic plasma concentrations, providing better clinical benefits [3].

Alkaloids are a class of naturally occurring nitrogen-containing compounds that have at least one nitrogen as a heteroatom, usually in a heterocyclic ring—with basic properties—and produce pronounced physiological responses. According to the biosynthetic pathway, alkaloids can be classified into, (1) true alkaloids that originate from amino acids and contain a nitrogen-based heterocyclic ring; (2) proto-alkaloids that also derive from amino acids, but do not contain a nitrogen moiety in a heterocyclic system; (3) pseudoalkaloids that do not originate from amino acids. Currently, more than 8000 natural compounds are classified as alkaloids [4,5].

Alkaloids are widely distributed in the plant kingdom, with some estimates that 25% of Gymnosperms and Angiosperms produce these metabolites. Moreover, alkaloids are present in plants from the families, Apocynaceae [6], Asteraceae [7], Papaveraceae [8], Rutaceae [9], Solanaceae [10], Erythroxylaceae [11], ans Fabaceae [12] among others.

Since the discovery of this class of natural products, several biological activities associated with alkaloids have been reported, including analgesic [13], antibacterial [14], antifungal [15], anti-inflammatory [16], anticancer [17], and antiviral [18] activity. Among the alkaloids that have antiviral activity, berberine has shown activity against the chikungunya virus, human cytomegalovirus (HCMV), and hepatitis C virus (HCV) [19,20,21], tomatidine against dengue virus (DV) [22], michellamine B against human immunodeficiency virus (HIV) [23], oxymatrine against influenza A virus [24], and palmatine against zika virus (ZV) [25]. In addition, plants rich in alkaloids also show antiviral action, such as the seeds of *Peganum harmala* L. that can inhibit influenza A virus [26] and root tubers of *Stephania cepharantha* Hayata that improve the survival of mice infected by herpes simplex virus type 1 (HSV1) [27].

Therefore, considering the therapeutic anti-viral potential, including anti-coronaviral, of this class of natural products, the aim of this study was to review the antiviral activity of alkaloids against coronaviruses. This study looked at the various alkaloids which showed in vitro and in vivo anti-coronavirus activity, with emphasis on human anti-coronaviral activity. In silico analysis was then performed to study the affinity of the eleven identified alkaloids—with anti-coronavirus activity—for binding to the receptor-binding domain of SARS-CoV-2 spike protein.

## 2. Results

**Figure 1:**
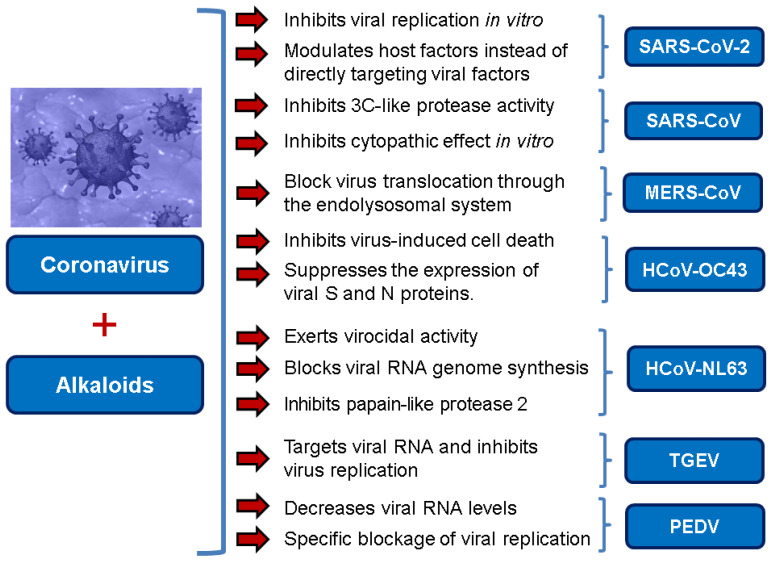
Main inhibitory actions of alkaloids against coronaviruses.

In recent times, natural alkaloids and alkaloid analogs have been studied extensively for their potent antioxidant and anti-inflammatory [28], as well as broad anti-viral properties [29]. In particular, with the current coronavirus pandemic in mind, alkaloids provide a rich source of important chemical compounds with great potential as novel anti-coronavirus agents (Figure 1) [30,31].

Homoharringtonine (HHT) is a plant-derived alkaloid extracted from some species of the *Cephalotaxus* genus [32,33]. HHT exhibits antiviral activity against a diverse range of viruses, including the varicella-zoster virus (VZV) [34], hepatitis B virus (HBV) [35], human echovirus 1 (HEV1) [36], vesicular stomatitis virus (VSV), Newcastle disease virus (NDV), and HSV1 [37] among others. Moreover, groups have reported that HHT shows strong anti-viral activity against diverse human and animal coronaviruses [37,38]. In a screen of 727 compounds in the NIH Clinical Collection small molecule database, HHT—with the lowest IC_50_ of the 84 compounds identified—was identified as the strongest inhibitor against various coronaviruses. In this study, HHT effectively inhibited coronavirus infection (a reduction in virus titer of ≥8 log_10_) by interfering with the coronavirus replication. The authors reported an IC_50_ of about 0.011 μM with a dose-dependent effect on virus inhibition [38]. In a later study by Dong and colleagues (2018), HHT was shown to effectively reduce porcine epidemic diarrhea virus (PEDV) viral load in infected cells and animals. The alkaloid was reported to provide optimal inhibitory activity, without death, at a dose of 0.05 mg/kg in piglets. Interestingly, HHT treatment effectively decreases mRNA levels of PEDV nucleocapsid in pig intestine and blood, indicating efficient inhibition of PEDV replication in vivo. Moreover, HHT-dosed piglets did not show a pathological change in tissues or symptoms of diarrhea and cachexia [37]. In a very recent study, HHT was reported to inhibit the in vitro replication of SARS-CoV-2. In Vero E6 cells, the alkaloid was reported to have a CC_50_ of 59.75 μM, and an EC_50_ of 2.55 and 2.14 μM for the reduction in infectious virus and reduction in viral RNA copy numbers, respectively [3]. The authors do not speculate on the mode of HHT action but it has previously been reported to be a protein synthesis inhibitor [33], so this might provide clues for future studies on the mode of action.

Lycorine is one of the major alkaloids isolated from the plant *Lycoris radiate*, a traditional Chinese medicinal herb [39]. This alkaloid possesses many diverse biological functions, including antiviral [39] and anti-inflammatory properties [40]. Lycorine has been reported to have antiviral action against many diverse viruses, including DV [41], ZV [42], poliovirus [43], HCV [44], enterovirus 71 (EV-71) and coxsackievirus A16 [45,46], avian influenza H5N1 virus [47], HSV1 [48], and bunyaviruses and Rift Valley fever virus (RVFV) [49]. Using a high throughput screening MTS assay to check for virus-induced cytopathic effect (CPE), Li and colleagues (2005) screened more than 200 anti-viral Chinese medicinal herb extracts for antiviral activity against the human SARS-CoV. In the end, they reported that lycorine—with an EC_50_ value of 15.7 ± 1.2 nM, a CC_50_ in Vero E6 and HepG2 cell lines of 14,980.0 ± 912.0 and 18,810.0 ± 1322.0 nM, respectively, and an SI value higher than 900 inhibits SARS-CoV replication in vitro, making it an ideal candidate as a new anti-SARS-CoV drug [30]. Unfortunately, this observation was made post the SARS-CoV outbreak, and could never be tested in an in vivo setting. Moreover, the authors did not speculate on the anti-SARS-CoV mode of action of lycorine. Based on the previous study by Li and colleagues (2005), Zhang and others (2020) tested the inhibitory effect of lycorine on SARS-CoV-2 in in vitro cell culture replication. Using a dose-dependent rescuing CPE assay, they reported that lycorine inhibits SARS-CoV-2 in vitro replication in a cell-independent manner [50]. While previous studies have reported that the antiviral action of lycorine is primarily through the suppression of viral RNA replication [41,45], by blocking the elongation of viral RNA translation during EV71 infection [45], others have reported that lycorine is able to stop the movement of influenza virus nucleoprotein from the nucleus [51] and is able to downregulate autophagy [46]. Based on these previous reports, Zhang and colleagues (2020) postulated that the anti-SARS-CoV-2 activity of lycorine is likely due to the alkaloid modulating host factors instead of directly targeting viral factors [50]; however, this needs to be verified in future studies.

Oxysophoridine belongs to the quinolizidine alkaloid group and is one of the alkaloids extracted from the Chinese medicinal plant *Sophora alopecuroides* and *Siphocampylus verticillatus* [52,53]. This alkaloid has various known pharmacological activities, especially in the field of oncology [54], and has anti-oxidative stress, strong anti-inflammatory, and anti-apoptosis properties [53,55,56]. Studies investigating the anti-viral properties of this alkaloid have not been very successful but a recent study has reported that SARS-CoV-2 replication is inhibited by oxysophoridine in cell culture (EC_50_ 0.18 μM and CC_50_ > 40 μM) [50]. Little-to-nothing is known about the anti-viral mode of action of oxysophoridine, however, and future studies are needed to determine how this alkaloid interferes with coronaviral replication.

The bis-benzylisoquinoline alkaloids tetrandrine (TET), fangchinoline (FAN), and cepharanthine (CEP) are major compounds in *Stephania tetrandra* and other related species of Menispermaceae; these alkaloids are known for their anticancer and anti-inflammatory activity [31]. The antiviral activities of these alkaloids have also been widely studied. The alkaloid fangchinoline exhibits antiviral activity against HIV1 in MT-4 and PM1 cells [57]. Some studies using cepharanthine showed that its antiviral action against HIV1 is by inhibiting the process of virus entry into cells, reducing the fluidity of the plasma membrane [58]; through a synergic effect with a derivative tetrahydrotetramethylnaphthalene results in inhibition of the proliferation of human T-lymphotropic virus type 1 (HTLV-1) [59]. On the other hand, tetrandrine attenuates the infection caused by the DV in human lung cells [60], inhibits the infection of human macrophages by EV [61], and inhibits HSVI-induced keratitis in mice [62]. Moreover, cepharanthine hydrochloride inhibits the replication of the lamivudine-resistant HBV [63]. A more recent study reported that several bisbenzylisoquinoline alkaloids block MERS-pseudovirus translocation through the endolysosomal system—which normally provides one pathway for cellular entry of MERS-CoV—by inhibiting NAADP-evoked Ca^2+^ release [64]. In addition, tetrandrine, fangchinoline, and cepharanthine inhibit HCoV-OC43-induced cell death in the early stage of infection in human lung cells and reduce virus replication by suppressing the expression of viral S and N proteins [31].

*Tylophora indica* and *Tylophora ovata* are used as traditional medicines in India [65] and China [66]. Tylophorine and tylophorine analogs, the natural products first isolated from the plant *T. indica*, are reported to have broad in vivo activity against many diseases, including inflammation [67]—one of the hallmarks of pathogenic coronavirus infection, and can inhibit global protein synthesis [68]. Interestingly, tylophorine has also been reported to inhibit the replication of the HCV [69]. More recent studies have shown that tylophorine-based compounds, whether extracted from plants or artificially synthesized, act as strong inhibitors of many coronaviruses including SARS-CoV, mouse hepatitis virus (MHV), and transmissible gastroenteritis virus (TGEV) [70,71,72]. One study described natural (tylophorine and tylophorinine) and synthetic tylophorine compounds as novel and potent anti-CoV agents for the treatment of TGEV and SARS-CoV infections. The authors reported EC_50_ values for the natural and synthetic tylophorine compounds ranging from 8 to 1468 nM and 5 to 340 nM, in ST and Vero 76 cells, respectively. Additionally, the tylophorine compounds showed strong anti-coronavirus replication activity, ultimately blocking virus-induced apoptosis and subsequent cytopathic effect in cells in vitro [71]. In a further study to understand the mode of action of tylophorine-based compounds, Yang and colleagues (2017) demonstrated that these compounds target viral RNA, thereby inhibiting TGEV replication. The authors further reported that tylophorine-based compounds act jointly with CYT387—a JAK family inhibitor—to exert comprehensive anti-TGEV activities. They concluded that the combination treatment, using a tylophorine compound and a JAK2 inhibitor, is more efficacious for the treatment of SARS-CoV or MERS-CoV than either treatment on its own [72].

*Isatis indigotica* is a traditional Chinese medicine used in clinical settings for its anti-viral properties in the treatment of diseases like influenza, hepatitis, and encephalitis [73,74], as well as inflammation [75]. In one study, indigo—a major compound of *I. indigotica* root extract—showed potent antiviral activity against Japanese encephalitis virus (JEV) in vitro replication in a dose-dependent manner. Moreover, time-of-addition assays showed that indigo exhibits a strong antiviral effect before or during infection, but not after viral cell entry [74]. Chang and colleagues (2012) hypothesized that this indicated that the antiviral mode of action of indigo is associated with the blocking of virus attachment to the cell receptor [74]. Interestingly, *I. indigotica* root was also frequently used for the prevention and treatment of SARS during the SARS-CoV outbreaks in China, Hong Kong, and Taiwan [76]. In fact, Lin and colleagues (2005) reported that both the *I. indigotica* root extract, as well as indigo, showed a significant inhibitory effect on SARS-CoV in the micromolar range. Similar to other *I. indigotica* root compounds, indigo inhibits the cleavage activities of the 3CLpro—a viral replication enzyme that mediates the proteolytic processing of coronavirus replicase polypeptides into functional proteins [77], in a dose-dependent manner. In the end, the authors reported indigo IC_50_ values for cell-free and cell-based assays of 300 μM and 752 μM, respectively; this is indicative of indigo acting as an efficient blocker 3CLpro functioning. Lastly, a CC_50_ of 7.4 mM in Vero cells was reported indicating that indigo is not toxic to Vero cells [76]. This contradicts the hypothesis for the mode of action for indigo proposed by Chang and colleagues (2012) but could merely be a function of the virus studied or the assays used. Further studies on different viruses, and in different systems, would be needed to elucidate the exact mode of action of indigo.

*Strobilanthes cusia* is a traditional medicine used in Myanmar, India, Thailand, and the southern parts of China [78]. Historically, the root has been used to treat influenza, encephalitis B, viral pneumonia, epidemic cerebrospinal meningitis, and mumps [79,80]. Tryptanthrin and indigodole B are two of the major compounds extracted from *S. cusia* leaves [81]. In a study by Tsai and colleagues (2020), these compounds showed strong antiviral activity in reducing both the CPE and virus yield (IC_50_ values are 1.52 μM and 2.60 μM for tryptanthrin and indigodole B, respectively) in HCoV-NL63-infected cells. In addition, strong viricidal activity was reported for tryptanthrin (IC_50_ = 0.06 μM) and indigodole B (IC_50_ = 2.09 μM). The authors identified tryptanthrin as the key antiviral compound of *S. cusia* leaf methanol extract with potent activity against HCoV-NL63 in a cell-type independent manner. Lastly, they reported that tryptanthrin interferes with the early and late stages of HCoV-NL63 in vitro replication, by blocking viral RNA synthesis as well as the activity of the papain-like protease 2 [82].

Due to the structural diversity of the alkaloids and the differences in the experimental protocols used by each research group in our selected articles, it is not easy to establish a relationship between chemical structure and anti-coronaviral activity of the alkaloids identified in our review. However, we do note that the identified alkaloids share some common chemical characteristics. Interestingly, the eleven alkaloids are highly oxygenated due to the presence of several functional groups, including methoxyls, hydroxyls, methylene dioxide, and carbonyls of ketones, esters, and amides. Also, the nitrogens found in these alkaloids are heterocyclic atoms and are sometimes in ring fusion, and most importantly, all the alkaloids are made up of polycyclic systems in their chemical structures. It is plausible that these common chemical structures contribute to the anti-coronavirus activity of these alkaloids.

**Figure 2:**
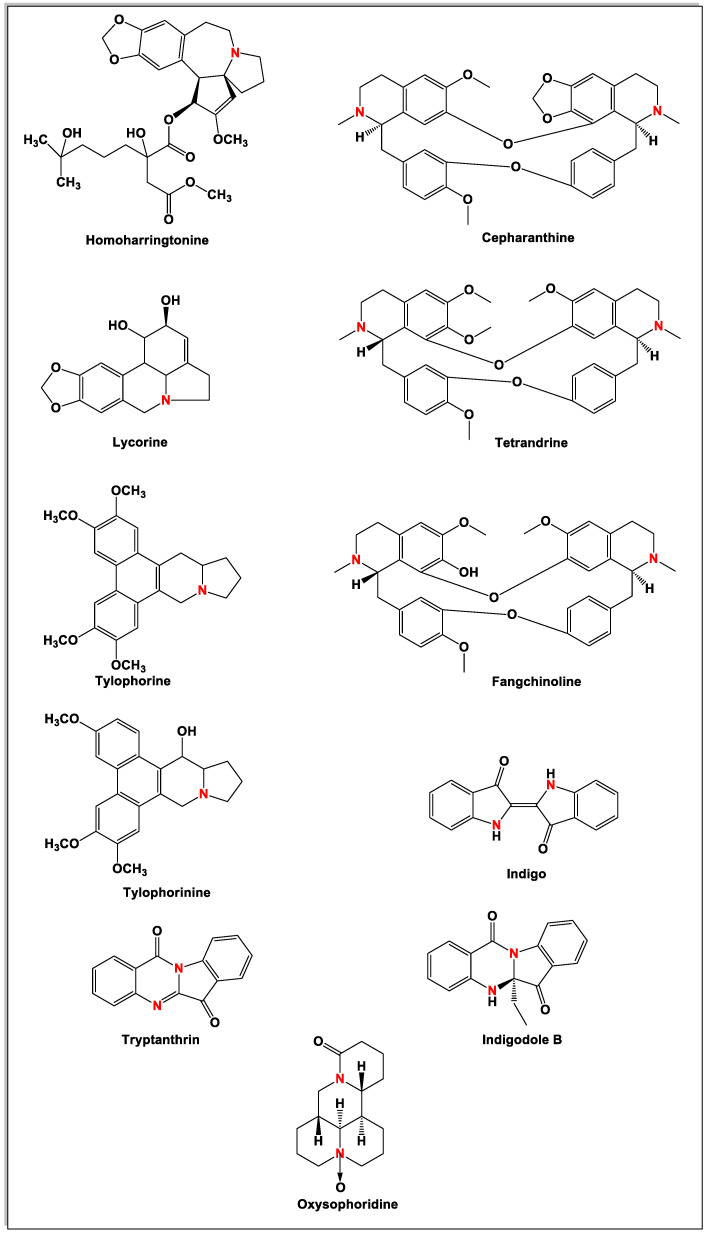
Chemical structures of bioactive alkaloids against coronavirus.

Current data demonstrate the therapeutic potential of these compounds against several viral diseases, including those related to coronaviruses (Table 1), as promising molecules for the study of new drug candidates against the COVID-19. Table 2 showed the activities of alkaloids against other viruses—besides coronaviruses—and the compounds are illustrated in Figure 2.

### Molecular Docking Study

SARS-CoV-2, a pandemic infectious disease resulting in COVID-19, is causing numerous health and economic problems globally. SARS-CoV-2 must bind to specific receptors on the host cell surface that allows virus entry into the host cell. SARS-CoV-2 attaches to angiotensin converting enzyme-2 (ACE2) receptors—the main receptor involved with viral entry—present on the surface of host cells, by anchoring the virus’ S1 subunit of the spike protein. The S1 subunit, which contains the receptor binding domain (RBD), is responsible for the high-affinity viral binding to ACE2 receptors [83]; this makes the SARS-CoV-2 S-RBD residues potential targets to control virus entry and infection.

In this study, the S1 subunit of SARS-COV-2 was targeted by an in silico approach to repurpose drug molecules that bind the S-protein and blocks its interaction with the ACE2 receptor, rendering it incapable of infecting a host cell. From a previous study [83], it was reported that several drugs were found from in a high-throughput virtual screening approach of FDA approved LOPAC library drugs to be active against the S-protein receptor of the virus. KT185, KT203, GSK1838705A, BMS195614, and RS504393 were identified to bind at the receptor binding site on the viral S-protein. So, in order to gain insights into the binding mode and crucial molecular interactions of the chosen alkaloids, molecular modeling simulation studies were performed with the S-RBD protein of SARSCOV-2 using Libdock protocol in Discovery Studio 2.5 Software. The analysis of their binding modes was performed to predict their biological activities and to achieve further insight into binding orientations and interactions. Alkaloids interacting with S1-RBD could potentially interfere with virus attachment to host receptors and, hence, inhibit virus entry into the host cell. Therefore, a molecular docking study was performed to identify and understand the interaction and binding affinity of these alkaloids with the S1-RBD of SARS-CoV-2.

The outcome of our docking study of the selected alkaloids and reported compounds with SARS-CoV-2 S1-RBD is presented in Table 3. Where the previously reported KT185, KT203, GSK1838705A, BMS195614, and RS504393 gave a libdock score of 95.9, 101.43, 72.7, 91.75, and 120 (Kcal/mol), respectively, the chosen alkaloids gave a libdock score ranging from 64.26 (Kcal/mol) to 109.11 (Kcal/mol). This finding indicated higher binding stability for alkaloids-S1-RDB complexes. In other words, S1-RDB favored interactions with most alkaloids, especially homoharringtonine which revealed the highest docking score. The interaction of homoharringtonine with S1-RBD involved three hydrogen bonds acceptor with Ser494, Ser494, and Gln 493 and two hydrogen bond donors with Tyr453 and Pro491. In addition, there were hydrophobic interactions with Leu455, Lys452, and Phe 497. Also, with cepharanthine, the high docking score (106.74 Kcal/mol) could be attributed to the formation of five hydrogen bond acceptors with Arg 454, Arg 457, Lys 458, Lys 458, and Ser 469 and one hydrogen bond donor with Gln474. All the docked structures showed similar binding modes in the binding region of S1-RBD of SARS-COV-2 and interaction with various amino acid residues, including the Leu455, Phe486, Asn487, Gln493, Ser494, Tyr495, and Gly496 [83]. This indicated that these compounds have a high probability of binding to the S-RBD protein of SARS-COV-2, preventing it from binding to the host cell.

## 3. Conclusions

Traditional herbal medicines and plant-based natural compounds are rich resources of new antiviral drugs. In fact, about 25% of commonly used medicines contain compounds isolated from plants. Many traditional herbal medicines possess antiviral activity against a plethora of viral strains, exerting their antiviral activity on the virus life cycle, including viral entry, replication, assembly, and release, and also the virus–host-specific interactions. Alkaloids have been reported as broad-spectrum inhibitors of animal and human coronaviruses. Whereas the mechanisms used by HHT, oxysophoridine and tylophorine, and tylophorine analogs to inhibit CoV replication are not known, lycorine modulates host factors to interfere with viral replication. On the other hand, tetrandrine, fangchinoline, and cepharanthine block virus translocation through the endolysosomal system or target viral RNA, inhibiting TGEV replication and act jointly with a JAK-family inhibitor for comprehensive anti-CoV activity. Indigo, on the other hand, inhibits the cleavage activities of the 3CLpro to interfere with virus replication. Lastly, tryptanthrin and indigodole B decrease virus yield and have potent viricidal activity. In particular, tryptanthrin was reported to block viral RNA genome synthesis as well as the activity of the papain-like protease 2 in HCoV-NL63-infected cells. As shown in the in silico simulations using biological targets related to SARS-CoV-2 and in some recent in vitro studies, the alkaloids provide an attractive prospect as anti-coronavirus drugs and warrants further investigation.

## 4. Methodology

The present study was carried out based on a search of the literature on alkaloids against human coronaviruses. A search of the literature was performed by using the scientific database PubMed, included studies published until April 2020, and used the following keywords: coronavirus, Human coronavirus-229E (229-E), Human coronavirus-NL63 (NL-63), Human coronavirus-OC43 (OC43), Human coronavirus-HKU1 (HKU1), SARS-CoV, MERS-CoV and SARS-CoV-2 (2019-nCoV or COVID-19), alkaloid, pyrrolidines, pyridines, tropanes, pyrrolizidines, isoquinolines, indoles, quinolines, quinazoline, *β*-carboline alkaloid, piperidine, and pyrrole. Articles published in languages other than English were excluded.

### Molecular Docking

The X-ray crystal structure of the S-RBD protein of SARS-COV-2 was obtained from the Protein Data Bank [www.rcsb.org] (PDB code: 6vw1) [84]. The protein structure was prepared using the default protein preparation tools integrated into the software. This was accomplished by adding hydrogen atoms to the amino acid residues, completing the missing residues, and applying force field parameters by using CHARMm forcefield [85]. All of the protein structures were minimized using 500 steps employing a SMART minimizer algorithm. Also, binding pockets, together with the surrounding amino acid residues, were identified. The ligands were prepared using the ligand preparation protocol of Accelrys Discovery Studio [86]. The ionization pH was adjusted to 7.4, hydrogen atoms were added and no isomers or tautomers were generated from the ligands. Docking was carried out using Libdock software [87] in the interface of Accelrys Discovery Studio 4.0 [86]. Ten docking poses were generated for each ligand docked and were then thoroughly inspected for proposing the best binding mode. The top-ranked poses were selected for analysis.

## Figures and Tables

**Table 1:** Examples of alkaloids active against coronaviruses.

Alkaloid	Coronavirus	Main Finding	Reference
Homoharringtonine (HHT)	SARS-CoV-2	EC_50_ 2.10 μM (reduction in viral copy number) EC_50_ 2.55 μM (reduction in infectious virus)	[3]
	MHV, BCoV-L9 and HECoV-4408	Inhibits viral replication IC_50_ 11 nM	[38]
	PEDV	IC_50_ 0.112 μM in Vero E6 cells Decreases viral RNA levels in vivo in piglets Specific blockage of viral replication	[37]
Lycorine	SARS-CoV	IC_50_ 15.7 nM	[30]
	SARS-CoV-2	Anti-CoV activity likely due to the lycorine modulating host factors instead of directly targeting viral factors	[50]
Oxysophoridine	SARS-CoV-2	EC_50_ 0.18 μM and CC_50_ > 40 μM	[50]
Tetrandrine, Fangchinoline, and Cepharanthine	MERS-CoV HCoV-OC43	Block MERS-pseudovirus translocation through the endolysosomal system Inhibited HCoV-OC43-induced cell death in the early stage of infection and reduced virus replication by suppressing the expression of viral S and N proteins.	[64] [31]
Tylophorine and Tylophorine analogs	SARS-CoV, MHV, and TGEV SARS-CoV, MERS-CoV, and TGEV	Anti-CoV replication activity; blocks virus-induced apoptosis and subsequent cytopathic effect in cells in vitro EC_50_ values for the natural and synthetic tylophorine compounds 8 to 1468 nM and 5 to 340 nM in ST and Vero 76 cells, respectively Targets viral RNA, thereby inhibiting TGEV replication Acts jointly with JAK family inhibitor for comprehensive anti-CoV	[71] [72]
Indigo	SARS-CoV	Inhibits the cleavage activities of the 3CLpro IC_50_ values for cell-free and cell-based assays of 300 μM and 752 μM, respectively	[76]
Tryptanthrin and Indigodole B	HCoV-NL63	Reduces viral yield: tryptanthrin (IC_50_ 1.52 μM); indigodole B (2.60 μM) Virucidal activity: tryptanthrin (IC_50_ = 0.06 μM); indigodole B (IC_50_ = 2.09 μM) Tryptanthrin blocks viral RNA genome synthesis and the activity of the papain-like protease 2	[82]

**Table 2:** Bioactivity of alkaloids against other viruses besides the coronavirus.

Alkaloid	Type of Virus/Cell Lines	Concentration/Dose	Antiviral Effect	Reference
Homoharringtonine	VZV/HFF cells HBV/HepG2 2.2.15 cells Echovirus 1/RPE cells VSV/HEK293T cells HSV1/Vero cells	10 ng/mL 0.03 μM 2 μM 0.12 μM 50 nM 139 nM	Down-regulation of VZV lytic gene transcripts Induces a 50% inhibition in HBsAg release Induces a 50% inhibition in HBV-DNA release Inhibit echovirus replication Inhibits the late stage of vesicular stomatitis virus replication Inhibits 50% of HSV1 replication	[34] [35] [36] [37]
Tylophorine	HCV	0.06 μM	Reduced replication of the HCV through inhibition of Cyclin A2	[69]
Fangchinoline	HIV1/MT-4, PM1, and human embryonic kidney cell line 293T cells	0.8 to 1.7 μM	Inhibits HIV1 replication by interfering with gp160 proteolytic processing	[57]
Lycorine	DV-2/A549 cells ZV/Vero, Huh7, and A549 cells RD cells HCV/Huh 7.5 cells Avian influenza H5N1 virus/GD178 and MDCK cells EV-71 H/Vero cells Coxsackievirus A16/Vero cells	0.8 μM 0.22 to 0.39 μM 0.058 μg/mL 0.316 μM 0.52 μM 2.04 μM 3.2 μM	Inhibits 50% of envelope protein production Inhibits 50% of ZV protein and envelope biosynthesis Inhibits 50% of virus replication Reduces HCV replication by 50% through suppresses the expression of Hsc70 Reduces the expression of viral proteins Inhibits 50% of virus replication Inhibits 50% of virus replication	[41] [42] [43] [44] [47] [46]
Indigo	JEV/BHK-21 cells	37.5 μg/ml	Inhibits 50% of virus replication	[74]
Tetrandrine	DV/A549 cells Ebolavirus/human macrophages HSV/BALB/c mice	1–10 μM 8 μM 15 mg/kg (i.p.)	Inhibited the DNA binding activity of NF-κB induced by DV and suppressed viral production Inhibited the infection of human macrophages by Ebolavirus Inhibited keratitis induced by HSV-I	[60] [61] [62]
Cepharanthine	HIV1/Molt-4 T cell line	5–20 μg/mL	Inhibited the entry of the virus by reducing the fluidity of the plasma membrane	[58]
Cepharanthine hydrochloride	HBV/HepG2 cells	2.14 μM 31.89 μM	Inhibited the virus replication Inhibited HBeAg production	[63]

**Table 3:** Libdock score of the anti-coronavirus alkaloids and the key amino acids involved in the H-bond interaction with the compounds.

Alkaloids	2D Diagram	Libdock Score (Kcal/mol)	Key Amino Acids
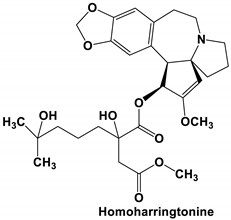	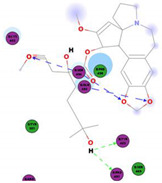	109.11	TYR 453 PRO 491 GLN 493 SER 494 SER 494
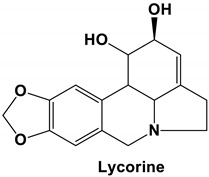	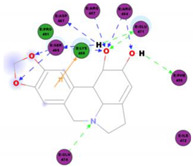	86.92	ARG 454 PHE 456 SER 469 Glu 471 Gln 474
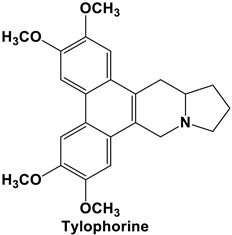	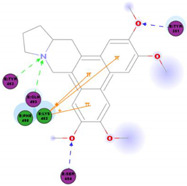	89.77	TYR 351 TYR 453 GLN 493 SER 494
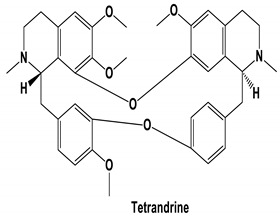	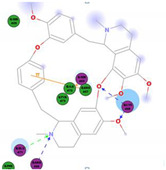	72.96	ARG 454 LYS 458 LYS 458 GLU 471
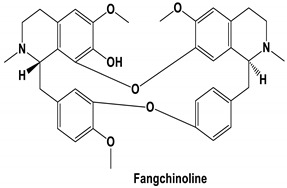	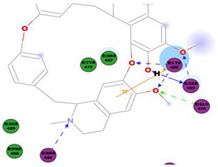	92.66	ARG 454 LYS 458 LYS 458 SER 469 GLN 474
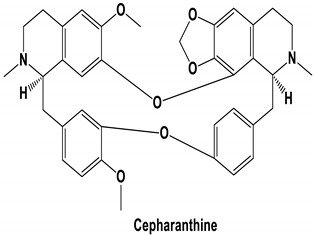	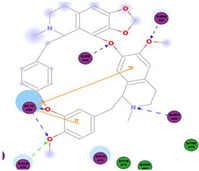	106.74	ARG 454 ARG 457 LYS 458 LYS 458 SER 469 GLN 474
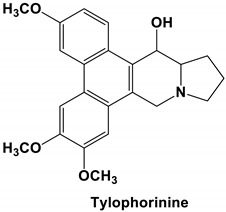	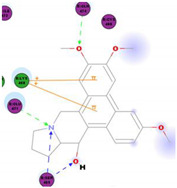	76.08	SER 469 SER 469 GLU 471 GLN 474
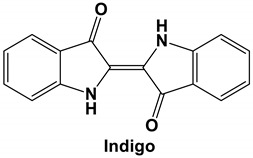	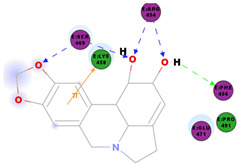	64.26	PHE 347 ASN 448 ASN 450
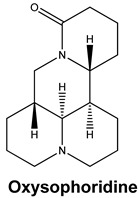	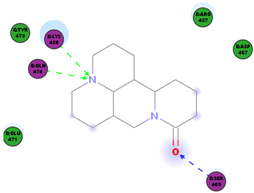	79.62	LYS 458 SER 469 GLN 474
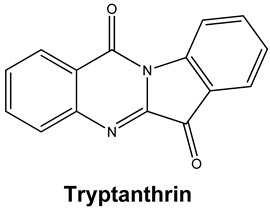	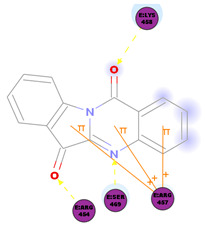	76.46	ARG 454 LYS 458 SER 469
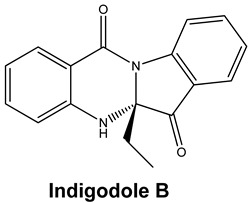	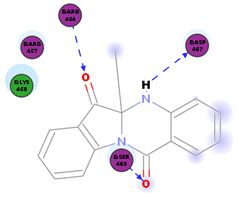	74.59	ARG 454 ASP 467 SER 469

## Abbreviations

229-E	Human coronavirus-229E
3CLpro	3C-like protease
BCoV-L9	Bovine coronavirus strain L9
CEP	Cepharanthine
CoVs	Coronaviruses
COVID-19	Coronavirus disease 2019
CPE	Cytopathic effect
DV	Dengue virus
EV	Ebolavirus
EV-71	Enterovirus 71
FAN	Fangchinoline
HBV	Hepatitis B virus
HCMV	Human cytomegalovirus
HCV	Hepatitis C virus
HECoV-4408	Human enteric coronavirus-4408
HEV1	Human echovirus 1
HHT	Homoharringtonine
HIV	Human immunodeficiency virus
HKU1	Human coronavirus-HKU1
HSV1	Human T-lymphotropic virus type
HTLV-1	Herpes simplex virus type 1
JEV	Japanese encephalitis virus
MERS-CoV	Middle east respiratory syndrome-coronavirus
MHV	Mouse hepatitis virus
NDV	Newcastle disease virus
NL63	Human coronavirus-NL63
OC43	Human coronavirus-OC43
PEDV	Porcine epidemic diarrhoea coronavirus
PLpro	Papain-like protease
RVFV	Rift Valley fever virus
SARS-CoV	Severe acute respiratory syndrome-coronavirus
SARS-CoV-2	Severe acute respiratory syndrome-coronavirus-2
S-RBD	Spike protein receptor-binding domain
TET	Bis-benzylisoquinoline alkaloids tetrandrine
TGEV	Transmissible gastroenteritis coronavirus
VSV	Vesicular stomatitis virus
VZV	Varicella-zoster virus
ZV	Zika virus
